# Reducing the burden of diarrhea among children under five years old: lessons learned from oral rehydration therapy corner program implementation in Northern Nigeria

**DOI:** 10.1186/s41043-015-0005-1

**Published:** 2015-05-01

**Authors:** Zulfiya Charyeva, Molly Cannon, Olugbenga Oguntunde, Aminu Magashi Garba, William Sambisa, Amos Paul Bassi, Mohammed Auwal Ibrahim, Saba’atu Elizabeth Danladi, Nurudeen Lawal

**Affiliations:** 1 Futures Group, 401 Meadowmont Village Circle, Chapel Hill, NC 27517 USA; 2 USAID/TSHIP Project, Abuja, Nigeria; 3 E4A\DFID Nigeria, Abuja, Nigeria; 4 JSI Inc., USAID/TSHIP Project, Abuja, Nigeria

**Keywords:** Diarrhea, Oral rehydration therapy corners, ORT, Nigeria, Child health

## Abstract

**Background:**

In Nigeria, diarrhea remains one of the leading causes of death among children under five years old. Oral Rehydration Therapy (ORT) corners were introduced to health facilities in Bauchi and Sokoto states to serve as points of treatment for sick children and equip caregivers with necessary skills in case management of diarrhea and diarrhea prevention.

**Objectives:**

The **operations research** study examined the effect of facility-based ORT corners on caregivers’ knowledge and skills in management of simple and moderate diarrhea at home, as well as caregivers’ and service providers’ perceived facilitators and barriers to utilization and delivering of ORT corner services. It also examined whether ORT activities were conducted according to the established protocols.

**Methods:**

This quantitative study relied on multiple sources of information to provide a complete picture of the current status of ORT corner services, namely surveys with ORT corner providers (N = 21), health facility providers (N = 23) and caregivers (N = 229), as well as a review of service statistics and health facility observations. Frequency distribution and binary analysis were conducted.

**Results:**

The study revealed that ORT corner users were more knowledgeable in diarrhea prevention and management and demonstrated better skills for managing diarrhea at home than ORT corner non-users. However, the percentage of knowledgeable ORT users is not optimal, and providers need to continue to work toward improving such knowledge. ORT corner providers identified a lack of supplies as the major barrier for providing services. Furthermore, the study revealed a lack of information, education and communication materials, supportive supervision, and protocols and guidelines for delivering ORT corner services, as well as inadequate documentation of services provided at ORT corners.

**Recommendations:**

Recommendations for ORT corners program planners and implementers include ensuring all ORT corners have oral rehydration salt (ORS) packages and salt, sugar, and zinc tablets in stock, a secured commodity supply chain to avoid stockouts, and adequate policies and procedures in place.

## Background

Recent mortality estimates among children under five years old show that just five countries account for 50 percent of global mortalities. Of those five, Nigeria accounts for 11 percent of all deaths [1]. Diarrheal disease is the third leading cause of infant and child mortality in developing countries; it is responsible for 11 percent of all under-five deaths worldwide [2] and claims the lives of 1.8 million children per year worldwide [3]. Despite a decrease in childhood diarrheal diseases from 4.6 million to 0.8 million over the last three decades, the number of diarrheal deaths remains unacceptably high [2,4,5]. In Nigeria, the prevalence of childhood diarrhea is 10 percent, with 26 percent of cases treated with oral rehydration salts (ORS) solution [6,7]. Diarrhea also accounts for more than 16 percent of deaths, estimated at 150,000 annually, among children under five years old [8,9].

For Nigeria to meet the Millennium Development Goal 4 (MDG4), the country must attain a two-thirds reduction in the under-five mortality rate from 230 deaths per 1000 live births in 1990 to 76 in 2015. The 2013 Nigeria Demographic and Health Survey [8] reported an under-five mortality rate of 128 deaths per 1000 live births which means an additional annual 20 percent reduction is needed by 2015 to meet the target. Healthy home practices and community-based care could save over 90,000 children a year in Nigeria [10].

The impact of diarrheal morbidity on disability-adjusted life years is likely to remain substantial even as diarrheal mortality diminishes following current trends [11-13]. Nutritional deficits caused by diarrhea can affect a child’s growth, fitness, cognition, and performance at school [11,13]. It is estimated that each diarrheal episode a child experiences in the months preceding the child’s second birthday increases the risk of being stunted by 5 percent [14]. Moreover, diarrheal illness in early childhood is associated with long-term adverse cognitive effects and decreased work productivity later in life [11].

Key measures to treat diarrhea in children include rehydration with intravenous fluids in case of severe dehydration, oral rehydration salt (ORS) solution for moderate or no dehydration, and zinc supplements to reduce the duration of a diarrhea episode and stool volume [15]. Reduction in diarrheal mortality in the past decades has been attributed largely to scaling up oral rehydration therapy (ORT), which consists of the oral administration of ORS and/or recommended home fluids including salt-sugar solution (SSS). ORT is judged to be the “most important medical advance of the 20th century” [16]. Research demonstrated that the therapy cures dehydration and prevents deaths [17-21]. ORT is a simple, highly effective, inexpensive, and technologically appropriate methodology [22]. However, currently only 39 percent of children with diarrhea in developing countries receive the recommended treatment [23].

Numerous studies and diarrheal prevention programs have identified health facility-based ORT corners as a cost-effective strategy to promote case management of diarrheal diseases in many developing countries [21,22]. Further, evidence shows that ORT corners reduce the number of diarrheal referrals and admissions in a hospital [24]. Research has also demonstrated that most children treated in ORT corners recover quickly after initial treatment with ORS solution and can be discharged and sent home thereafter [21].

We conducted **an operations research** study to examine the effect of facility-based ORT corners on caregivers’ knowledge and skills in management of simple and moderate diarrhea at home, as well as caregivers’ and service providers’ perceived facilitators and barriers to utilization and delivering of ORT corner services in Northern Nigeria. **The study was part of an ongoing strategy for setting up ORT corners in Bauchi and Sokoto states in northern Nigeria.**

## Methods

### Study context, population and sites

In view of the high burden of diarrhea among children under five years old in Nigeria, the Targeted States High Impact Project introduced 225 ORT corners between March 2010 and August 2011 in Bauchi (n = 105) and Sokoto (n = 120) states. An ORT corner is a designated area within a health facility where caregivers can receive practical demonstrations on how to prepare ORS solutions and access lifesaving rehydration for sick children under the supervision of a healthcare provider. The ORT corners also build the capacity of caregivers with preventive and curative skills for home management of diarrhea. The ORT corners were located in health facilities to serve as direct or referred points of service for the treatment of diarrhea among children under the age of five. The study was conducted in 21 randomly selected communities with ORT corners (10 in Bauchi, 11 in Sokoto) between February and April 2012. The research questions of the study included: What are caregivers’ knowledge and skills in management of simple and moderate diarrhea at home? To what extent are ORT corners able to maintain ORT-related supplies? What type of counseling is provided at ORT corners and in the health facilities? What are the policies, support, and supervision at the ORT corners? What are perceived facilitators and barriers to utilization of ORT corners?

### Study design

**This was an operations research study that involved quantitative data collection from multiple sources,** including: ORT corner users (N = 110 caregivers with children under five years old accessing ORT corners services in the last three months); ORT corner non-users (N = 119 caregivers with children under five years old who had diarrhea in the last three months but did not visit a service provider and did not visit an ORT corner for treatment); ORT corner providers (N = 21); and health facility providers (N = 23). Additional data were gathered through a review of service statistics at ORT corners (N = 21) and health facilities (N = 23), and from observations at ORT corners (N = 21). The study team applied systematic random sampling to select two Local Government Areas (LGAs) in each of the three senatorial zones in Sokoto and Bauchi states (12 total), and then randomly selected ORT corners in each of the selected LGAs (24 total). ORT corner users (N = 110) were randomly selected from the list of users in the selected 24 ORT corners. Convenience sampling was applied to select ORT corner providers, health facility providers, and ORT corner non-users. Based on providers’ availability, we surveyed one provider from each of the selected ORT corners and one provider from each of the health facilities where ORT corners were located. The ORT corner users and non-users came from the same communities. Community leaders helped data collectors identify ORT corner non-users in their communities. **Before commencement of the surveys, written informed consent was obtained from each participant who was able to write and sign their name. Data collectors read the consent form and documented verbal consent for those participants who could not write.**

### Ethical clearance

The study protocol and all instruments were submitted to and approved by the Bauchi and Sokoto State Health Research Ethics Committees and the Health Media Lab Corporation in Washington, DC.

#### Data analysis

The study team triangulated data from the different data sources to provide a full picture of the functionality of the ORT corners in diarrhea management and prevention in the selected ORT corners and health facilities. We compared the responses of ORT corner users and non-users to examine the effect of ORT corners on caregivers’ knowledge and skills in managing simple and moderate diarrhea at home. Frequency distribution and binary analysis were conducted. With regard to desk review of service statistics, repeated monthly quantitative data analysis was conducted for detecting trends in selected indicators through generating relevant frequency tables and charts.

## Results

### Study participants

All ORT corner user caregivers were mothers of their children. On average, respondents had four children. The majority of respondents were in the 20–39 age range. More than ninety percent respondents were married and Muslim. Respondents came from different education and work backgrounds, and more than half lacked a formal education and worked as petty traders.

Demographic characteristics of the ORT corner non-users were similar to those of the ORT corner users, with the exception of occupation and number of children. There were more full-time housewives and fewer government employees in this group compared to the group of ORT corner users. On average, ORT corner non-users had fewer children than ORT corner users (Table 1).

**Table 1:** **Demographic characteristics of ORT corner users (N = 110) and non-users (N = 119)**

**Characteristics**	**ORT corner users, n(%)**	**ORT corner non-users, n(%)**	**p value**
*Age:*			**0.4905**
18-19 years	9(8)	11(9)	
20-29 years	48(44)	63(53)	
30-39 years	33(39)	35(30)	
40 and over	10(9)	9(8)	
*Education:*			**0.1048**
No formal education	59(54)	61(52)	
Primary or secondary education	33(30)	34(29)	
Tertiary education	10(9)	4(3)	
Other	8(7)	18(15)	
*Occupation:*			0.0016*
Housewives	35(32)	58(48)	
Petty traders	58(53)	55(46)	
Government employee	15(13)	2(2)	
Students	0(0)	2(2)	
Other	2(2)	2(2)	
Religion*: Muslim*	106(96)	112(94)	**0.4271**
Marital Status*: Married*	101(92)	113(95)	**0.3373**
Number of children, Mean	4.2	3.3	0.0027*

*p < 0.05.

The type of ORT corner providers and health facility providers were similar. Of the 21 ORT corner providers and 23 health facility providers, almost half (48 percent and 52 percent, respectively) were community health extension workers (CHEWs), and 29 percent and 35 percent, respectively were nurses. The remaining 24 percent of ORT corner providers were environmental health assistants and technicians. Two doctors and one environmental health technician also served as health facility providers. Of the 21 ORT corners, 7 (33 percent) were located in primary health centers (PHCs), 6 (29 percent) were located in hospitals, 5 (24 percent) in maternal and child health centers (MCHs), and 3 (14 percent) in dispensaries (14 percent).

### Knowledge and practices of ORT corner users and non-users on diarrheal disease prevention and treatment

ORT corner users indicated they would offer more liquids to their child during diarrhea than ORT corner non-users would (p < 0.0001), demonstrating better knowledge in preventing dehydration than ORT corner non-users. Almost 20 percent of non-users could not explain how much liquid they would offer if their child had diarrhea (Table 2).

**Table 2:** **Amount of liquid and food given to children under 5 years old during a diarrheal episode as reported by ORT corner users (N = 110) and non-users (N = 116)**

	**ORT corner users, n(%)**	**ORT corner non-users, n(%)**
Amount of liquid given:**		
About the same	**13(12)**	**6(5)**
More	**71(65)**	**56(50)**
Somewhat less	**13(12)**	**9(8)**
Much less	**13(12)**	**22(20)**
Don’t Know	**0(0)**	**20(17)**
Amount of food given:**		
About the same	**16(15)**	**11(10)**
More	**45(41)**	**15(13)**
Somewhat less	**15(14)**	**22(19)**
Much less	**28(26)**	**52(45)**
Stopped food	**3(3)**	**1(1)**
Don’t know	**3(3)**	**15(13)**

**p < 0.01.

ORT corner users demonstrated better knowledge in preventing malnutrition than ORT corner non-users (p < 0.0001). More ORT corner users than non-ORT corner users indicated they would give the same amount of food to their child during diarrhea or offer more food to their sick children. Despite their demonstrating better knowledge than non-users, almost 40 percent of the ORT corner users still indicated that they would give their children less, much less than usual, or no food at all during diarrhea. Sixty-five percent of the ORT corner non-users indicated such feeding practices (Table 2).

ORT corner users’ reported use of an ORS package to manage diarrhea was double that of non-users (78 percent, n = 86 and 39 percent, n = 47, respectively, p < 0.0001). Similarly, users’ ability to correctly describe how to prepare ORS at home was more than three times that of non-users (62 percent, n = 68 and 19 percent, n = 22 respectively, p < 0.0001).

ORT corner users’ reported knowledge of how to prepare salt-sugar solution (SSS) at home was more than double that of ORT corner non-users (74 percent, n = 81 and 30 percent, n = 35, respectively, p < 0.0001). Of the individuals who reported knowing how to prepare SSS, 67 percent of ORT corner users (n = 54) described it correctly, compared to 40 percent of non-users (n = 14), (p = 0.007).).

Both ORT corner users and non-users indicated use of ORS packages as their first choice for treating their children, followed by SSS. However, the percentages of caregivers using ORS and SSS were much higher among ORT corner users (60 percent using ORS and 37 percent using SSS) than non-users (40 percent, p = 0.003 and 15 percent, p = 0.0001, respectively).

When asked to select ways to prevent diarrhea from a list of options, ORT corner users demonstrated a much better knowledge on prevention than non-users (p < 0.05) (Table 3). However, just 49 percent of ORT corner users indicated hand-washing at key moments and 29 percent indicated safe excreta disposal as ways to prevent diarrhea.

**Table 3:** **Knowledge of diarrhea preventing measures and danger signs of diarrheal sickness, ORT corner users (N = 110) and non-users (N = 119)**

	**ORT corner users, n(%)**	**ORT corner non-users, n(%)**	**p value**
*Ways to prevent diarrhea:*			
Use of clean water for drinking and cooking	**69(63)**	**53(45)**	0.0058**
Preventing insects contamination	**71(65)**	**50(42)**	0.0006**
Hand-washing at key moments	**54(49)**	**42(35)**	0.0345*
Safe excreta disposal	**32(29)**	**15(13)**	0.0020**
Don’t know	**9(8)**	**31(26)**	0.0004**
*Danger signs of diarrheal sickness:*			
Not able to drink or drinking poorly	**50(46)**	**36(30)**	0.0176*
Vomits everything	**50(46)**	**31(26)**	0.0022**
Convulsions	**8(7)**	**4(3)**	0.1845
Abnormally sleepy	**28(26)**	**30(25)**	0.9661
Restless or irritable	**49(45)**	**61(51)**	0.3095
Blood in stool	**13(12)**	**5(4)**	0.0324*

*p < 0.05; **p < 0.01.

The knowledge of danger signs for when to visit a service provider immediately was higher among ORT corner users than among non-users in three categories (not able to drink or drinking poorly, vomits everything, and blood in stool, p < 0.05). Despite this, and the fact that response options to the questions on danger signs were provided for respondents, very few knew that blood in stool and convulsions were danger signs. Overall, the percentage of ORT corner users indicating associated symptoms as danger signs were in a low range, from 7 percent to 46 percent (Table 3).

Thirty percent of ORT corner users (n = 33) reported their child having had diarrhea since their last visit for diarrhea to the ORT corner. Among these, almost half (49 percent) gave ORS solution to their children, more than a third gave them SSS (36 percent), and 15 percent took their child to a health facility.

### Factors related to caregivers accessing ORT corners and health facilities

More than 90 percent of all respondents reported the need to get permission from their husband to attend the health clinic when their child has diarrhea and needs to see a service provider. Other influential people included mothers-in-law (10 percent), fathers-in-law (8.3 percent), and mothers (5 percent) of the caregivers.

The 119 ORT corner non-users listed various reasons for not seeing a service provider when their child has diarrhea. These include their perception of diarrhea as being related to teething (38 percent), that every newborn experiences diarrhea and that the child will recover even without treatment, it is just a matter of time (16 percent), diarrhea as inevitable in infants and young children (11 percent), and diarrhea being a sign of survival among newborns (8 percent). Other factors preventing non-users from visiting a service provider include perceived high cost of treatment (13 percent) and transportation challenges (15 percent).

ORT corner non-users reported the following advantages to seeing a service provider when their child has a diarrheal bout: to get treatment (68 percent), to make a child feel better (29 percent), for child to recover faster (19 percent), and to get prescriptions for medicine (24 percent).

### Status of ORT corner facilities and support for ORT corner service providers

Data from ORT corner provider interviews and observations indicate that registers were available in 14 of 21 ORT corners. All of these registers included information on the patient’s date, name, age, serial number, sex, treatment given, and address information. However, registers did not always have complete information: 5 did not have dehydration status, 12 did not include information on consultation topic, and 13 were missing information on the duration of stay. According to service statistics, the average monthly number of users per ORT corner was 15. Of the 21 ORT corners, 9 (43 percent) recorded information on the number of ORT corner users who were sent home with necessary advice, children treated at the corner, or children admitted to a hospital for the last eight months. Four of these nine corners (19 percent) had such information for some, but not all, of the eight month period. The remaining 11 corners did not record these service statistics.

In terms of availability of information, education, and communication (IEC) materials, 17 of the 21 ORT corners had posters on the walls. Brochures and leaflets were much less common, with two of the twenty-one (10 percent) ORT corners possessing them.

On the day of the survey, of the 23 health care facilities, 22 percent used piped water, 52 percent used water from a protected well, and 17 percent used water from an unprotected well. In almost three-quarters of the health facilities, the water source was located within the health facility compound (70 percent, n = 16).

According to surveys with ORT corner providers and observations conducted in the corners, the most recent version of written guidelines/protocols for delivering ORT corner services was not available in nine ORT corners (43 percent). Eight corners (38 percent) had them available and the protocols were seen by interviewers. One corner reported having the recent protocols but the interviewers did not see them.

Approximately half of ORT corner providers reported receiving a refresher course in diarrhea management (52 percent, n = 11). On average, providers received this training 12 months prior to the survey (range is 3–23 months). However, all but 10 percent indicated needing additional refresher training on diarrhea management. Providers also expressed the need for additional training on diarrhea prevention and control (52 percent, n = 11), communication and counseling (33 percent, n = 7), and diarrhea treatment (33 percent, n = 7).

The majority of the providers (67 percent, n = 14) reported receiving monitoring visits from a supervisor and that these visits took place either monthly (n = 3), quarterly (n = 4), or bi-annually (n = 7). Most of the visits included on-the-job training (n = 7), re-supply of materials (n = 6), assistance with completing registers (n = 6), and providing general support (n = 4). Three of the fourteen who were visited reported receiving a supervision visit report or recommendation. Recommendations included improve reporting of stockouts, keep ORT corners clean, and submit requests for IEC materials.

### Status of health facilities and training received by health facility providers

Because ORT corners are located in health facilities and rely on referrals from health facility providers, it is important to understand how the health facilities work with regard to diarrheal disease treatment.

According to interviews with health facility providers and based on the research team’s observations, registers were available in 12 of the 23 health facilities. However, of the 12 registers available, only three provided information on child’s dehydration status, two recorded information on the type of treatment provided and none included information on referrals to the ORT corner. Despite the missing documentation on ORT corner referrals, health facility providers estimated that they refer about 60 percent of children with diarrhea to ORT corners (mean is 57 percent, SD is 41.8, range is 0–100). This statistic could not be verified in this study.

Fewer than half of health facility providers (39 percent, n = 9) reported ever receiving any training in diarrhea management. On average, their training took place 21 months prior to the survey (range is 3–50 months).

### Services provided at ORT corners

All ORT corner providers reported providing counseling services to a caregiver of a child with diarrhea in the last month. Group counseling was provided in most cases (62 percent, n = 13), followed by individual counseling (38 percent, n = 8). Between 57 percent to 86 percent of respondents reported always demonstrating ORS preparation; giving ORS packages to caregivers; and explaining how to prepare ORS/SSS, how often to give it to a child, and when to throw it away. However, only four of the respondents reported providing zinc tablets to a child with diarrhea, and fewer than half of the providers (44 percent, n = 7) always explained the dosage and duration of treatment to clients (Table 4). ORT corner providers reported providing the three key messages about home treatment of diarrhea (give more fluid, continue feeding, and danger signs for when to see a service provider immediately) to 70 percent of the caregivers bringing children to the ORT corners.

**Table 4:** **Activities implemented by ORT corner providers (N = 21) as reported by providers**

**Activity**	**Frequency of implementation (n,%)**			
	**Always**	**Occasionally**	**Rarely**	**Never**
Provided ORS preparation demonstration, n = 21	14(67)	7(33)	0(0)	0(0)
Gave ORS packages to caregivers, n = 21	12(57)	5(24)	2(10)	2(10)
Explained how to prepare ORS/SSS, how often give it to a child, and when to throw it away always, n = 21	18(86)	3(14)	0(0)	0(0)
Gave zinc tablets to a child with diarrhea, n = 20	4(20)	5(25)	2(10)	9(45)
Explained the dosage of zinc tablets and duration of treatment, n = 16	7(44)	3(19)	1(6)	5(31)

**Of the 110 ORT corner users, 81 percent reported that ORT corner providers discussed diarrhea-related issues during their last visit to the ORT corner. Slightly more than one-third of ORT corner users had questions about the management of diarrhea that they wanted to ask service providers during their last visit. In nearly all of those cases (98 percent), users were allowed to ask those questions, and 95 percent received satisfactory responses from the ORT corner provider. The total time ORT corner users spent at the ORT corner varied, but on average was 41 minutes, with half of that time spent waiting to see a provider (mean 20 minutes, range 2–60 minutes, SD = 15.45), and the other half receiving services (mean 21 minutes, range 2–180 minutes, SD = 25). On average, 12 of the 21 minutes were spent talking to a service provider (range 2–60 minutes, SD = 9).**

### Availability of commodities at ORT corners

Regarding the availability of commodities, 12 of the 21 ORT corner providers reported that ORS packages were available on the day of the survey; and one-third or fewer had salt, sugar and zinc tablets available (Table 5). Providers reported stockouts of all supplies in the last three months, most frequently for zinc tablets, salt and sugar. ORT corner providers also described delays in replacing sugar or salt in the case of stockouts, with some saying it takes up to a week (29 percent, n = 6), a month (24 percent, n = 5), or up to six months (14 percent, n = 3).

**Table 5:** **Availability of commodities at ORT corners as reported by service providers (N = 21)**

**Supplies**	**Availability**		
	**Usually available**	**Available today**	**Stock out last 3 months**
	**n(%)**	**n(%)**	**n(%)**
ORS packages	14(67)	12(57)	6(29)
Zinc tablets	3(14)	1(5)	13(62)
Sugar	6(29)	7(33)	10(48)
Salt	7(33)	6(29)	10(48)

Findings from ORT corner users reinforce ORT provider findings. Of the 110 ORT corner users, 66 percent reported receiving ORS packages during their last visit to the ORT corner. Half of ORT corner users indicated absence of one or more ORT-related supplies during their last visit at the ORT corners, specifically, no zinc tablets (86 percent), ORS packages (46 percent), salt (25 percent), and sugar (27 percent) at the ORT corners.

## Discussion

Our study suggests that ORT corners play an important role in diarrhea management. The study demonstrates that ORT corner users are more knowledgeable about the amount of liquids and food to give to a sick child, ORS and SSS preparation, danger signs for when to visit a service provider immediately, and ways to prevent diarrhea than ORT corner non-users. Sixty-five percent of ORT corner users in our sample reported offering more liquids to a child with diarrhea compared to only ten percent of caregivers nationally, reported by 2013 DHS [8]. Furthermore, ORT corner users demonstrated having skills for managing diarrhea at home. For example, among ORT corner users who reported their child having diarrhea since their last visit to an ORT corner, the majority gave ORS and SSS (49 percent and 36 percent respectively), and only 15 percent took their children to a health facility. However, despite ORT corner users having better knowledge of key diarrheal prevention messages and danger signs for when to visit a service provider immediately than non-users, the percentage of knowledgeable ORT users is still relatively low. ORT corner and health facility providers in both states need to do more to improve such knowledge. This also stresses the need for more community based health education regarding diarrheal disease.

The study also identified barriers for seeking medical treatment that could be addressed through education and awareness-raising. For example, our sample of ORT corners’ non-users beliefs regarding inevitability of diarrhea, its relation to teething, and not considering it as a life threatening disease deterred them from seeking medical attention. Health communication messages need be developed to correct these misconceptions and encourage their use of health care and ORT corner services.

Our study confirms previous studies’ findings that husbands have a strong influence in health care-seeking behavior of female caregivers [25,26]. Consequently, there is a need to sensitize and train this group regarding the advantages of health services for their children.

This study collected data about policies and procedures at health facilities. Client satisfaction with ORT corner services was high, indicating that staff were friendly and able to answer clients’ questions, and that waiting times were not too long. However, several areas for improvement regarding policies and procedures at ORT corners need to be addressed. These include improving availability of IEC materials, providing supportive supervision, improving registers, keeping and recording more information on services provided by ORT corners, and ensuring availability and adherence to protocols and guidelines for delivering ORT corner services.

The study also looked at issues of availability of materials at ORT corners and stockouts and found that supply of salt, sugar, ORS packages and zinc tablets in both states was insufficient, a situation that needs to be addressed urgently. Overall, ORT corner providers identified a lack of supplies as the major barrier for providing services to the users. Furthermore, with more than half of ORT corner users indicating absence of the ORT-related supplies during their last visit to ORT corners, there is a threat of a decrease in demand for ORT services in the future unless measures are taken immediately to ensure continuous availability of supplies [27].

This study has several limitations. First, though the results of the study may be used to inform interventions in Bauchi and Sokoto states, we need to exercise caution generalizing findings and recommendations to the other states of Nigeria. Also, we were not able to answer some of the research questions because service statistics data in most of ORT corners and health facilities were not available or were incomplete. In the surveys with the ORT corners and health facilities providers as well as with the caregivers, we rely on self-reported data, which may be subject to social desirability bias. We tried to minimize biases by training data collectors on proper survey techniques and ensuring respondents of data confidentiality. Finally, while we examined the issue of access to safe water at ORT corners, we did not focus on environmental issues such as safe water and sanitation in the communities overall. In addition to ensuring effective work of ORT corners, these broader environmental issues would need to be a focus of diarrhea prevention programs [28].

Among study strengths is that the study findings are based on use of surveys with multiple groups of providers and service users as well as review of service statistics. This is the first operations research study to inform future interventions at ORT corners in Bauchi and Sokoto states, Nigeria, and the study findings can inform the design and implementation of ORT corner programs in other settings. Based on this study’s findings, we recommend that ORT corners program planners and implementers train both ORT corner and health facility providers to counsel all caregivers on diarrhea management at home and danger signs for when to visit a service provider immediately; taking steps to ensure all ORT corners have ORS packages, salt, sugar, and zinc tablets in stock and a secured commodity chain to avoid stockout; and ensuring ORT corners and health facilities have adequate policies and procedures in place.

## Conclusions

ORT corners play an important role in diarrhea management. ORT corners program planners and implementers need to ensure all ORT corners have a secured commodity supply chain to avoid stockouts, and adequate policies and procedures in place.
